# Myogenic Differentiation of iPS Cells Shows Different Efficiency in Simultaneous Comparison of Protocols

**DOI:** 10.3390/cells10071671

**Published:** 2021-07-02

**Authors:** Aleksandra Ulman, Marta Kot, Klaudia Skrzypek, Barbara Szewczyk, Marcin Majka

**Affiliations:** 1 Department of Transplantation, Medical College, Jagiellonian University, 31-008 Krakow, Poland; aleksandra.ulman@doctoral.uj.edu.pl (A.U.); marta.kot@uj.edu.pl (M.K.); klaudia.skrzypek@uj.edu.pl (K.S.); barbara.szewczyk@med.uni-rostock.de (B.S.); 2 Department of Transplantation, Institute of Pediatrics, Faculty of Medicine, Jagiellonian University Medical College, 30-663 Krakow, Poland

**Keywords:** myogenesis, skeletal muscle, differentiation, induced pluripotent stem cells (iPS), myogenic factors, CD56

## Abstract

Induced pluripotent stem (iPS) cells constitute a perfect tool to study human embryo development processes such as myogenesis, thanks to their ability to differentiate into three germ layers. Currently, many protocols to obtain myogenic cells have been described in the literature. They differ in many aspects, such as media components, including signaling modulators, feeder layer constituents, and duration of culture. In our study, we compared three different myogenic differentiation protocols to verify, side by side, their efficiency. Protocol I was based on embryonic bodies differentiation induction, ITS addition, and selection with adhesion to collagen I type. Protocol II was based on strong myogenic induction at the embryonic bodies step with BIO, forskolin, and bFGF, whereas cells in Protocol III were cultured in monolayers in three special media, leading to WNT activation and TGF-β and BMP signaling inhibition. Myogenic induction was confirmed by the hierarchical expression of myogenic regulatory factors MYF5, MYOD, MYF6 and MYOG, as well as the expression of myotubes markers MYH3 and MYH2, in each protocol. Our results revealed that Protocol III is the most efficient in obtaining myogenic cells. Furthermore, our results indicated that CD56 is not a specific marker for the evaluation of myogenic differentiation.

## 1. Introduction

Induced pluripotent stem cells obtained from somatic cells by reprogramming resemble embryonic stem cells according to morphology, proliferation, telomerase activity, gene expression profile, and ability to differentiate into three germ layers. By mimicking processes that take place during human embryo development, they constitute a powerful tool for deciphering mechanisms regulating embryogenesis [1].

Skeletal muscles are formed during embryonal development in the process known as myogenesis. They are characterized as multinucleated striated fibers responsible for motion and support [2]. Myogenesis is a complicated multistep process regulated by the interplay between intrinsic and extrinsic regulatory factors [3]. First, embryonal stem cells are directed into mesenchymal fate, which is controlled by WNT [4], FGF [5,6], and BMP [7] signaling pathways. After paraxial mesoderm generation, cells undergo segmentation and form somites. At this stage, cells are not committed to a specific lineage. The compartmentalization of somites leads to dermomyotome formation and, further, to a myotome creation as a result of cell delamination [8].

The degree of cell specification into myogenic fate is reflected by the expression of specific genes—myogenic regulatory factors (MRFs). Myogenic factor 5 (MYF5) is activated first, then myogenic differentiation 1 (MYOD) and myogenic factor 6 (MYF6) are induced. Myogenin (MYOG) is a terminal factor that controls the differentiation of myoblasts into myocytes [2]. Myocyte fusion into multinucleated myotubes and their further maturation results in skeletal muscle fiber formation with the characteristic expression of myosin heavy chain isoforms, embryonal MYH3 at the beginning and, later, adult MYH2 [9,10].

The need to study myogenesis arises because of diseases associated with the musculoskeletal system, such as dystrophies [11] or sarcomas [12,13]. A better understanding of the mechanisms driving skeletal muscle formation may underlie future treatment methods, for instance, the generation of patient-specific muscle stem cells, namely, satellite cells characterized by PAX7 expression [14]. They constitute a regenerative pool for skeletal muscle; however, their isolation [15] and propagation are inefficient [16].

Currently, in the literature, many different protocols for skeletal muscle differentiation are available. They can be divided into transgene-based methods with forced exogenous expression of myogenic factors [17,18] and transgene-free methods in which particular myogenesis stages are recapitulated by the addition of inhibitor cocktails that mimic the embryonal environment. In our study, we focus on transgene-free methods that differ according to the duration, applied factors, type of culture, and stromal equivalent [19,20,21,22,23]. However, all of them struggle with similar imperfections such as overall low efficiency, unspecific differentiation [24], and long cell culture [25].

Here, we undertake the challenge to compare three promising protocols for skeletal muscle cell generation in order to evaluate the most efficient one. In the first protocol (Protocol I), the induction of differentiation is initiated by the creation of three-dimensional cell aggregates called embryonic bodies (EBs). Next, the outgrown cells, cultured in serum-free conditions with ITS (insulin–transferrin–selenium), are selected by adhesion to collagen type I and then propagated in a medium supplemented with horse serum (HS) and fetal bovine serum (FBS) [26]. Protocol II is set to strong myogenic induction at the beginning, during EB formation, by BIO, forskolin, and bFGF (basic fibroblast growth factor) addition. In the next step, cells proliferate and mature in HS-supplemented medium [27]. The last protocol (Protocol III) is performed in monolayer culture and is based on three different media, all of which lead to mesoderm differentiation as a result of WNT activation and TGF-β inhibition, myotome formation by the inhibition of both TGF-β and BMP, and maturation [28]. Our results show that Protocol III, with the monolayer cell culture and defined signaling obtained by the addition of many factors, results in the most efficient production of myofibers.

## 2. Materials and Methods

### 2.1. Cell Culture

A human protein-induced iPS cell line generated from fibroblasts was purchased from SBI System Biosciences, Palo Alto, CA, USA. The cells were cultured as described previously [29,30,31] in serum-free iPS cell medium containing DMEM/F12 supplemented with 20% KSR, 2 mM Glutamax, 100 µM non-essential amino acids, 10 ng/mL bFGF, 100 U/mL penicillin/streptomycin (all from Thermo Fisher Scientific, Waltham, MA, USA), and 100 µM β-mercaptoethanol (Sigma-Aldrich, Saint Louis, MO, USA) with daily medium change on 0.1% gelatin-coated (Sigma-Aldrich) dishes with a feeder layer of mouse embryonic fibroblasts (MEFs) inactivated with mitomycin C (Sigma-Aldrich) at 37 °C, 5% CO_2_, 5% O_2_, and 95% humidity. Cells were passaged with Accutase cell detachment solution (BioLegend, San Diego, CA, USA) and seeded on new dishes with density 1:4–1:10 in medium with 10 µM ROCK inhibitor Y-27632 (Sigma-Aldrich). In feeder-free conditions, cells were cultured on dishes coated with growth-factor-reduced Matrigel (Corning, New York, NY, USA) in StemMACS iPS-Brew XF (Miltenyi Biotec, Bergisch Gladbach, Germany). MEFs (AMSBIO, Abingdon, UK) were cultured in Dulbecco’s modified Eagle’s medium (DMEM) containing 4.5 g/L glucose (Lonza, Basel, Switzerland) supplemented with 10% *v*/*v* FBS (Eurx, Gdansk, Poland), 2 mM L-glutamine and 100 U/mL penicillin/streptomycin antibiotics solution (all from Thermo Fisher Scientific). Cell lines were routinely tested for mycoplasma contamination using a MycoAlert™ Mycoplasma Detection Kit (Lonza).

### 2.2. Protocol I Differentiation

As was previously described by Awaya et al. [26], iPS cells were collected with Accutase and transferred in a 1:2 ratio to non-adherent plates with standard iPS medium without bFGF to form EBs. EBs were cultured for 7 days, with the medium changed every other day. Subsequently, EBs were plated in a 1:3 ratio on adherent dishes coated with 0.1% gelatin in DMEM medium supplemented with 10% FBS, and 2 mM L-glutamine. The next day, the medium was replaced with ITS medium—DMEM low glucose (Lonza), ITS-X supplement (Thermo Fisher Scientific), 100 µM β-mercaptoethanol, 100 µM non-essential amino acids, L-glutamine, and 100 U/mL penicillin/streptomycin—and changed every other day. After 14 days, the cells that migrated from EBs were dissociated with 0.05% trypsin and plated on a dish coated with type I collagen (Sigma-Aldrich), according to vendor’s instruction, and cultured in SkIM (skeletal muscle induction) medium—DMEM high glucose (Thermo Fisher Scientific), 10% FBS, 5% horse serum (Thermo Fisher Scientific), 100 µM β-mercaptoethanol, 100 µM non-essential amino acids, L-glutamine, and 100 U/mL penicillin/streptomycin—at the density of 3000 cells/cm^2^. The medium was changed every day for 28 days. Next, the medium was replaced with ITS medium for the last 14 days and changed every other day.

### 2.3. Protocol II Differentiation

As it was previously described by Xu et al. [27], iPS cells were passaged with 1 U/mL dispase (Thermo Fisher Scientific) and transferred to suspension culture in myogenesis-promoting medium (STEMdiff APEL 2 Medium, STEMCELL Technologies, Vancouver, BC, Canada), 10 ng/mL bFGF, 0.5 µM BIO (Sigma-Aldrich), 20 µM forskolin (Sigma-Aldrich), and 100 U/mL penicillin/streptomycin) for EB formation. The medium was changed every other day. After 7 days, cells were seeded on a dish coated with Matrigel (BD) in a 1:3 ratio in DMEM high glucose medium (Thermo Fisher Scientific) supplemented with 2% horse serum (Thermo Fisher Scientific) and 100 U/mL penicillin/streptomycin and cultured for further 35 days with daily medium change.

### 2.4. Protocol III Differentiation

As was previously described by Wu et al. [28], cells were dissociated with Accutase and seeded on Matrigel-coated plates at a density of 10,000 cells/cm^2^ in MDM I medium: IMDM (Thermo Fisher Scientific) supplemented with 5% horse serum, 3 mM CHIR99021 (Tocris Bioscience, Bristol, UK), 2 mM SB431542 (Selleck Chemical LLC, Houston, TX, USA), 10 ng/mL hr-EGF (epidermal growth factor; PeproTech Inc., London, UK), 10 mg/mL insulin (Sigma-Aldrich), 0.4 µg/mL dexamethasone (Sigma-Aldrich), and 200 mM L-ascorbic acid (Sigma-Aldrich) with medium changed every other day. After 4 days, cells were harvested using Accutase and seeded on Matrigel-coated plates at a density of 10,000 cells/cm^2^ in MDM II medium consisting of IMDM supplemented with 5% horse serum, 10 mg/mL insulin (Sigma-Aldrich), 10 ng/mL hr-EGF (R&D Systems, Minneapolis, MN, USA), 20 ng/mL hr-HGF (hepatocyte growth factor; PeproTech Inc.), 20 ng/mL hr-FGF (PeproTech Inc.), 10 ng/mL IGF-1 (insulin-like growth factor-1; PeproTech Inc.), 2 mM SB431542 (Selleck Chemical LLC), 0.5 mM LDN193189 (Stemgent, Cambridge, MA, USA), and 200 mM L-ascorbic acid (Sigma-Aldrich), replaced every other day. On day 15, the cells were harvested with Accutase and seeded without sorting on a Matrigel-coated dish (300,000/well of a 6-well plate) and cultured till day 21. On day 21, the medium was changed to MDM III (IMDM, 15% knockout serum replacement, and 10 ng/mL IGF-1), and the cells were cultured for another 2 days.

### 2.5. Flow Cytometry

To analyze the expression of the surface antigens, the differentiated cells were harvested with Accutase and incubated with: APC-conjugated antibody against CD56 or with APC-conjugated antibody against CD10 and PE-conjugated antibody against CD24 (all from Becton Dickinson, Franklin Lakes, NJ, USA) for 30 min at 4 °C in darkness. Corresponding isotype antibodies were used as the control to exclude non-specific binding. The results were analyzed using Attune NxT Software v2.2 (Thermo Fisher Scientific, Waltham, MA, USA) on an Attune Nxt Flow cytometer (Thermo Fisher Scientific, Waltham, MA, USA).

### 2.6. Extraction of RNA and Reverse Transcription Reaction

RNA was isolated using a GeneMATRIX Universal RNA/miRNA Purification Kit (EURx), according to the manufacturer’s instruction. RT-PCR with random primers (Promega, Madison, WI, USA) and Moloney murine leukemia virus MMLV reverse transcriptase (Promega) was performed according to the vendor’s protocol.

### 2.7. Real-Time PCR

Gene expression levels were evaluated using quantitative real-time PCR analysis. The following human TaqMAN probes were used (Applied Biosystems, Foster City, CA, USA) with the Quant Studio 7 Real-Time PCR System (Applied Biosystems): GAPDH (Hs99999905_m1), PAX7 (Hs00242962_m1), MYF5 (Hs00271574_m1), MYOD (Hs00159528_m1), MYF6 (Hs01547104_g1), MYOGENIN (Hs01032275_m1), MYH2 (Hs00430042_m1), MYH3 (Hs01074230_m1), DES (Hs00157258_m1), and Blank qPCR Master Mix (2×) (EURx). The mRNA expression levels for all samples were normalized to the levels of housekeeping gene GAPDH using the 2^−ΔCt^ method, which allowed us to calculate the relative expression of genes.

### 2.8. Visualization of Cellular Morphology

The morphology of the cells was analyzed using an Olympus IX70 microscope (Olympus Corporation, Tokyo, Japan) and a Canon EOS1100D digital photo camera (Canon Inc., Tokyo, Japan).

### 2.9. Wright Staining

Cell culture medium was aspirated, and cells were washed with PBS. Next, 0.5 mL per well of Wright’s dye (Sigma-Aldrich) was added to a 12-well plate. After 7 min of incubation at room temperature, an equal amount of Wright’s staining buffer was added. Cells were incubated for another 7 min at room temperature and washed twice with water.

### 2.10. Immunofluorescence Staining

Cells were washed with PBS (Eurx) and fixed in 4% paraformaldehyde at room temperature for 20 min. Next, after PBS wash, cells were permeabilized with 0.01% Triton X-100 at room temperature for 5 min, again washed with PBS, and blocked in 3% bovine serum albumin (BSA, Sigma-Aldrich) at room temperature for 30 min. Then, incubation with primary antibody mouse anti-vimentin antibody (V9):sc-6260 (Santa Cruz Biotechnology Inc., Santa Cruz, CA, USA), 1:250, in 4 °C overnight in 3% BSA was performed. Subsequently, after washing with PBS, cells were incubated with secondary antibody rabbit anti-mouse IgG, Alexa Fluor 555 (Thermo Fisher Scientific), diluted 1:250, and with Hoechst33342 (Sigma-Aldrich) in 3% BSA. After 1 h of incubation, cells were washed with PBS.

### 2.11. Western Blot

The nuclear and cytoplasmic fractions of proteins were isolated with a Nuclear Extract Kit (Active Motif, La Hulpe, Belgium) according to the manufacturer’s instruction. The protein concentration was measured with a Bradford reagent (BioRad, Hercules, CA, USA) using a Tecan Spark 10 M microplate reader (Tecan Trading AG, Männedorf, Switzerland). Proteins were separated by electrophoresis in a 12% resolving sodium dodecyl sulfate–PAGE gel, and the fractionated proteins were transferred into a PVDF membrane (BioRad). The blot was incubated with 1% BSA for 1 h and then overnight with primary antibodies, followed by incubation with secondary antibodies. Chemiluminescent signals were developed using SuperSignal™ West Pico PLUS chemiluminescent substrate (Thermo Scientific) using a Gel Doc imaging system (BioRad). Western blotting was performed using anti-GAPDH rabbit mAb (14C10; #2118; Cell Signaling Technology, Leiden, The Netherlands) and anti-myogenin mouse mAb (sc-12732; Santa Cruz Biotechnology Inc.). Secondary anti-rabbit and anti-mouse antibodies were conjugated with horseradish peroxidase (HRP, Santa Cruz Biotechnology Inc.). Western blot results are presented as representative images of three independent biological experiments.

### 2.12. Myotubes Formation

To quantify myotube formation after differentiation, we calculated nuclei numbers and the multinucleated cells from 2 (Protocol I) and 3 (Protocols II and III) biological replicates. The result presents the number of multinucleated cells as a percentage of total nuclei. The results are presented as means with SEM.

### 2.13. Statistical Analysis

Unless stated otherwise, the results show the mean ± the standard error of the mean (SEM) of at least 2 to 5 independent experiments. Statistical analysis was performed by one-way analysis of variance (ANOVA) with Tuckey’s or Dunnett’s post-test or Student’s *t*-test using GraphPad Prism software. Differences with a *p*-value less than 0.05 were considered statistically significant. For myotube formation, a statistical analysis chi-square test was performed using GraphPad Prism software.

## 3. Results

### 3.1. The Difference in Three Protocols of iPS Differentiation into Skeletal Muscles

**Figure 1:**
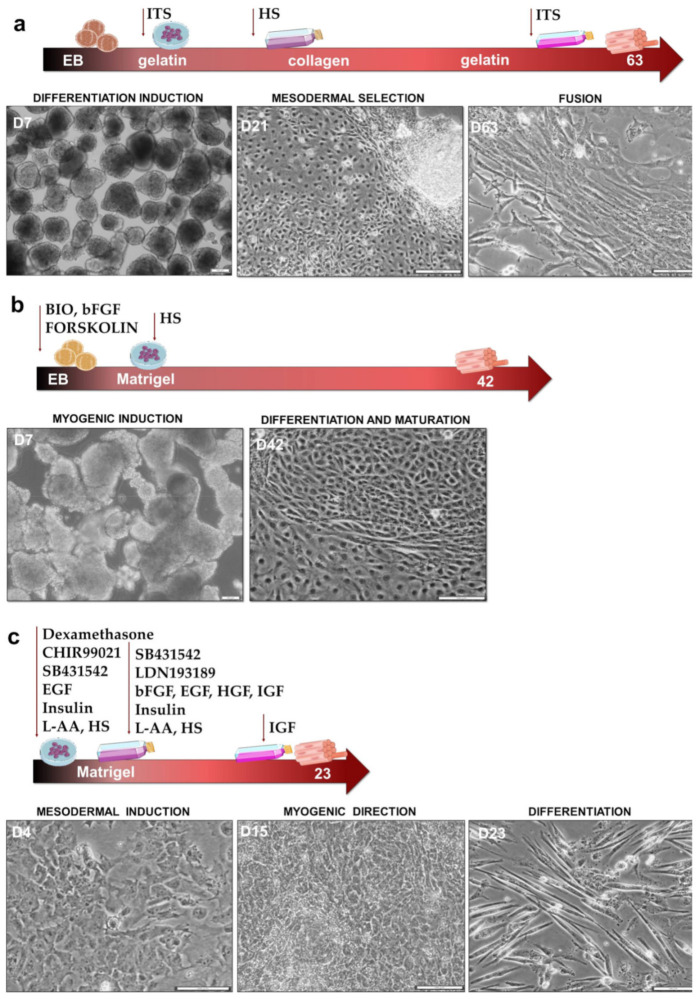
Schematic representation of the key steps, signaling modulators, and duration of particular protocols of differentiation into skeletal muscle with corresponding cell morphology changes. Scale bar represents 50 μm. (**a**) Protocol I; (**b**) Protocol II; (**c**) Protocol III.

Based on the literature review, three protocols, named in this study as Protocol I [26], II [27], and III [28], have been chosen as potentially promising to recreate myogenesis in vitro. Schematic presentation of the key steps in the particular protocols is visualized in Figure 1.

Protocol I (Figure 1a) is based on the induction of differentiation by EB formation. After 7 days of culture, formed aggregates are plated on a gelatin-coated dish in serum-free medium supplemented with ITS, which enables FBS reduction [26]. On day 21, outgrown cells are dissociated and subjected to selective expansion on collagen-type-I-coated dishes with medium containing both FBS and HS for 4 weeks. It is the stage of myogenic mesenchymal cell expansion. To force the final maturation of the cells, they are put back into serum-free medium with ITS until day 63. Protocol II [27] characterizes a very strong myogenic induction at the EB formation step by supplementing serum-free medium with bFGF, BIO, and forskolin (Figure 1b). The addition of bFGF minimizes premature differentiation [22,32]. BIO is a GSK-3 (glycogen synthase kinase-3) inhibitor that activates WNT signaling to promote mesoderm differentiation [33], whereas adenylyl cyclase activator forskolin promotes satellite cell proliferation [27]. After 7 days, cells are plated on a Matrigel-coated dish for further differentiation and maturation in a medium with 2% HS for an additional 35 days. The shortest protocol, called Protocol III, is based on three different media and does not include the EB formation step [28]. The first medium directs iPS cells into mesenchymal PAX7^+^ cells through WNT activation by GSK-3 inhibitor CHIR99021 and TGF-β inhibition with SB431542 [28]. After 4 days, the cells are seeded on a Matrigel-coated dish in the second medium, where they differentiate into MYF5^+^ cells by TGF-β and BMP inhibition with LDN193189. On the 15th day of the protocol, the cells are dissociated and again plated on a Matrigel-coated dish in the second medium. The final maturation of cells in the third medium takes two days and is supported by IGF-1, which promotes terminal differentiation [32]. The morphology of the cells in the different stages of the protocols is visualized in Figure 1.

### 3.2. Protocol I Based on the Selective Expansion of Myogenic Mesenchymal Cells on Collagen I

**Figure 2:**
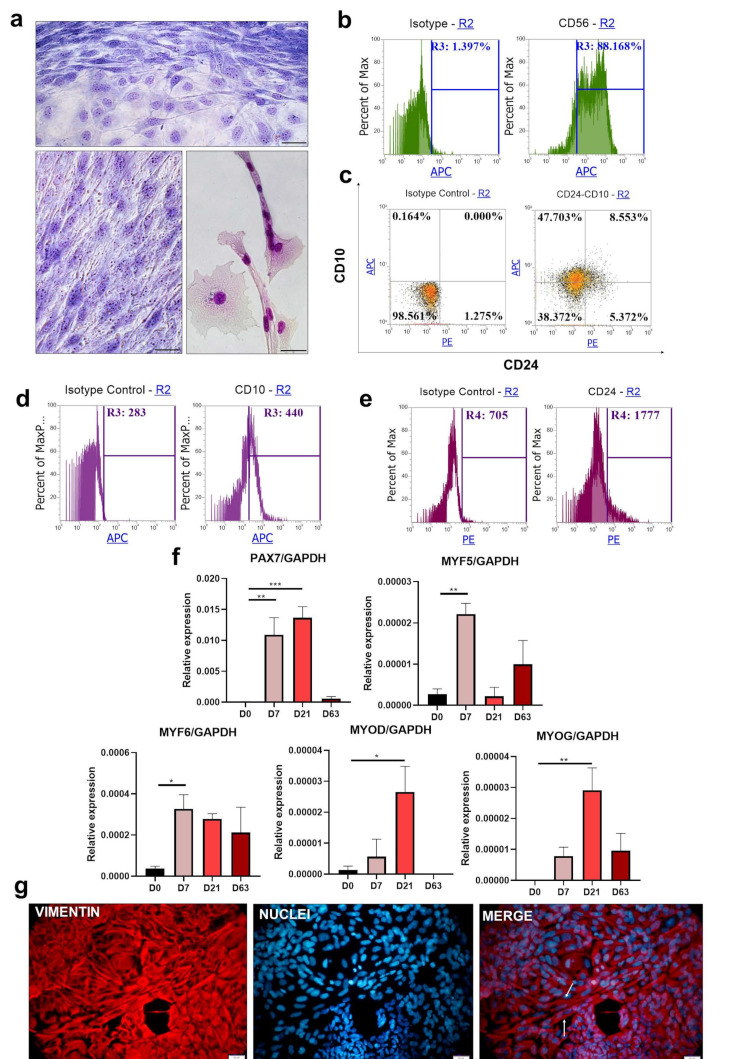
Differentiation of iPS cells into skeletal muscle cells with Protocol I: (**a**) morphology of the cells visualized with Wright’s staining on day 63 of the experiment. Scale bar represents 50 μm. (**b**) representative results of flow cytometry analysis of CD56 expression on day 63; (**c**) double staining for CD10 and CD24 expression analyzed with flow cytometry on day 63, representative result; (**d**) analysis of the total CD10^+^ mean fluorescence intensity on day 63, representative result; (**e**) analysis of the total CD24^+^ mean fluorescence intensity on day 63, representative result; (**f**) qPCR analysis of myogenic genes expression during the differentiation, *n* = 2, * *p* < 0.05, ** *p* < 0.01; *** *p* < 0.001 (**g**) vimentin and cell nucleus immunofluorescence staining at the end of the experiment. Scale bar represents 50 μm; white arrows indicate cell fusion.

iPS cells were differentiated according to Protocol I [26]. After 63 days of differentiation, the cells acquired a mesenchymal phenotype, with the presence of spindle-like-shaped cells (Figure 2a). Single multinucleated myotubes, generated as a result of cell fusion, could be detected.

Analysis of the expression of the CD56 molecule (Figure 2b) reveals the presence of two populations; nevertheless, most of the cells (almost 88%) had CD56 present on their surface. Double staining for CD10 and CD24 was proposed previously as an effective way to isolate myogenic cell populations from differentiated populations [28]. CD10 presence was confirmed on human primary myoblast and satellite cells. Furthermore, cells characterized by the expression patterns of CD10^+^ and CD24^−^ were proven as those that contribute to the latter myotubes’ population. Therefore, CD10^−^ and CD24^+^, CD10^−^ and CD24^−^ as well as CD10^+^ and CD24^+^ populations represent unspecific differentiation during the protocol. Almost 48% of the cells represented a specific myogenic population—positive for CD10 and negative for CD24 (Figure 2c). The negative cells constituted a group with 38% of all cells. CD24 was expressed by less than 20% of the total population. Analysis of total CD10 and CD24 mean fluorescence intensity in the population showed MFI 440 and 1777, respectively (Figure 2d,e). To verify the induction of myogenesis, we evaluated the expression of the associated genes at consecutive time points on days 0, 7, 21, and 63 (Figure 2f). Expression of *PAX7* and *MYF5* was expected at the beginning of the differentiation process [20,34]. *PAX7* strong induction of expression was detected from day 7, and it was further increased on day 21. *MYF5* and *MYF6* were significantly induced on day 7, and then their transcript levels were downregulated. *MYOD* transcript level was the highest on the 21st Day of the differentiation, and *MYOG* upregulation was also induced from this day. Immunofluorescence staining for vimentin (Figure 2g) shows the heterogeneous composition of the obtained cell population, which is indicated by the presence of a variety of different cell shapes. The analysis confirms the ubiquitous mesenchymal character of the generated cells. Long, multinucleated spindle-like cells were detected. These results prove that the cells undergoing differentiation with Protocol I can be directed into myogenic precursors, although with very low overall efficiency, as suggested by relative myogenic gene expression.

### 3.3. Protocol II—Based on Early Myogenic Induction with BIO, bFGF, and Forskolin

**Figure 3:**
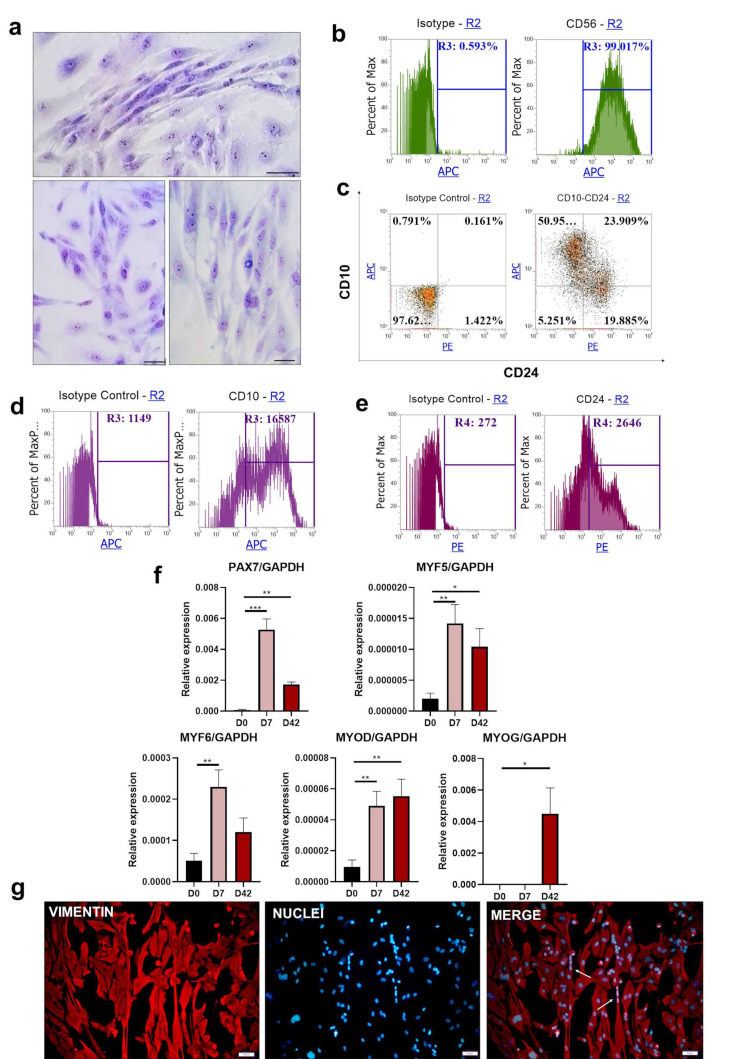
Differentiation of iPS cells into skeletal muscles with Protocol II: (**a**) morphology of the cells visualized with Wright’s staining on day 42 of the experiment. Scale bar represents 50 μm. (**b**) representative results of flow cytometry analysis of CD56 expression on day 42; (**c**) double staining for CD10 and CD24 expression analyzed with flow cytometry on day 42, representative result; (**d**) analysis of the total CD10^+^ mean fluorescence intensity on day 42, representative result; (**e**) analysis of the total CD24^+^ mean fluorescence intensity on day 42, representative result; (**f**) qPCR analysis of myogenic genes expression during the differentiation, *n* = 3 or more 4, * *p* < 0.05, ** *p* < 0.01, *** *p* < 0.001; (**g**) vimentin and cell nucleus immunofluorescence staining at the end of the experiment. Scale bar represents 50 μm; white arrows indicate cell fusion.

iPS cells were differentiated according to Protocol II [27] for 42 days. The morphology of the cells after differentiation represented a heterogeneous population (Figure 3a). Nevertheless, polarized and spindle-like shaped cells were visible.

Despite the heterogeneous morphology of the cells, the whole population displayed the expression of CD56 on the surface (Figure 3b). The CD10-positive fraction constituted a separate population that represented 51% of all cells (Figure 3c). CD10^+^ and CD24^+^ cells, as well as CD10^−^ and CD24^+^ cells, created the second main population. Only 5.25% of the cells were negative for CD10 and CD24. Histogram representation of mean fluorescence intensity indicated MFI = 16587 for total CD10^+^ population and MFI = 2646 for total CD24^+^ population (Figure 3d,e). An analysis of gene expression levels reflected a typical myogenic pattern, with significant upregulation of *PAX7*, *MYF5*, and *MYF6* on day 7; both *MYOD* and *MYOG* had high expression on day 42 (Figure 3f). Vimentin and cell nucleus staining confirmed the mesenchymal phenotype of the cells and visualized single-fused myotubes (Figure 3g). However, similar to the results from Protocol I, they were scattered among many other cell types.

### 3.4. Protocol III—Based on WNT, BMP, and TGF-β Signaling Pathways as an Efficient Way to Generate Skeletal Muscles

**Figure 4:**
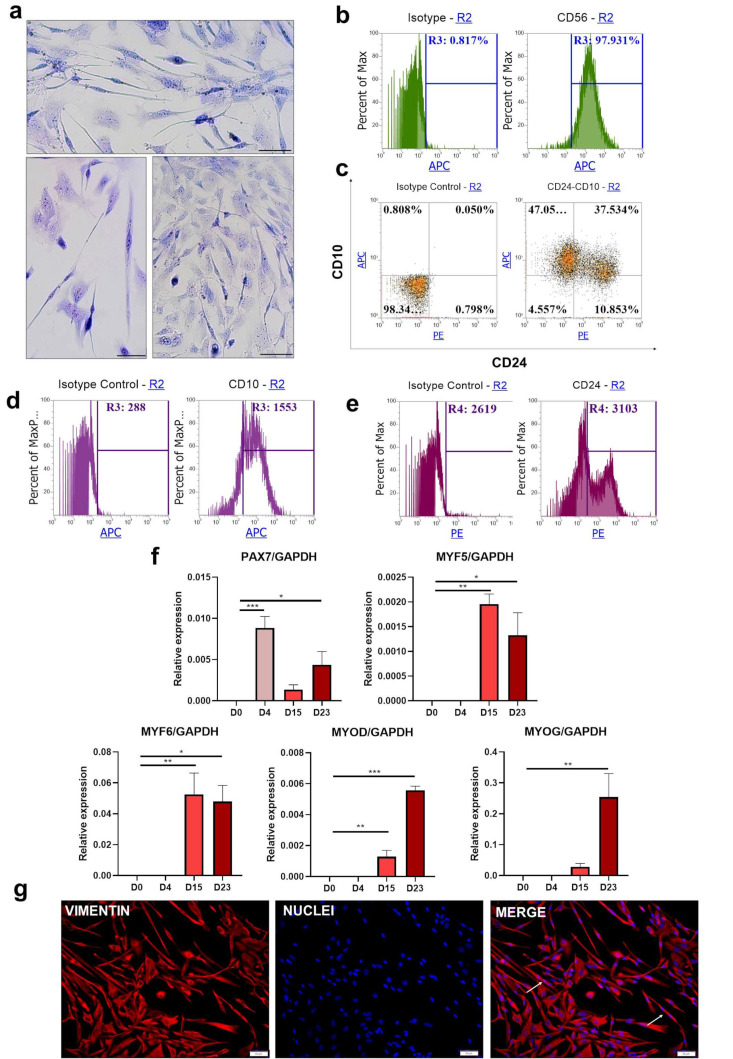
Differentiation of iPS cells into skeletal muscle cells with Protocol III: (**a**) morphology of the cells visualized with Wright’s staining on day 23 of the experiment. Scale bar represents 50 μm. (**b**) representative results of flow cytometry analysis of CD56 expression on day 23; (**c**) double staining for CD10 and CD24 expression analyzed with flow cytometry on day 23, representative result; (**d**) analysis of the total CD10^+^ mean fluorescence intensity on day 23, representative result; (**e**) analysis of the total CD24^+^ mean fluorescence intensity on day 23, representative result; (**f**) qPCR analysis of myogenic gene expression during the differentiation, *n* = 3 or 4, * *p* < 0.05, ** *p* < 0.01, *** *p* < 0.001; (**g**) vimentin and cell nucleus immunofluorescence staining at the end of the experiment. Scale bar represents 50 μm; white arrows indicate cell fusion.

iPS cells were differentiated according to Protocol III [28] for 23 days but without the sorting step. Wright’s staining on day 23 of the experiment (Figure 4a) revealed the presence of elongated, spindle-shaped cells. Moreover, it was possible to distinguish multinucleated cells; however, the heterogeneity of the population was visible.

Flow cytometry evaluation of CD56 expression (Figure 4b) revealed that the CD56 molecule was present on almost all cells despite the heterogeneous morphology of the cells visualized in Figure 4a. Analysis of CD10 and CD24 staining (Figure 4c) revealed the presence of two main populations, CD10^+^, CD24^−^ (47%) and CD10^+^, CD24^+^ (37%). Negative population and CD24^+^ together represented less than 15% of all cells. Total CD10^+^ population was characterized by MFI = 1553, whereas for total CD24^+^, MFI = 3103 (Figure 4d,e). Transcript analysis again confirmed the hierarchical expression of muscle-associated factors (Figure 4f) during differentiation. Induction of *PAX7* on day 4 was followed by *MYF5* and *MYF6* upregulation on ay 15. *MYOD* and *MYOG* reach the highest level on the last day of the protocol. Immunofluorescence staining for vimentin indicated the mesenchymal phenotype of all differentiated cells (Figure 4g). Importantly, the majority of the cells display spindle-like shaped morphology, and multinucleated cells were visualized as well.

### 3.5. Comparison of Different Protocols of Myogenic Differentiation

As presented in Figure 1, the tested protocols differ according to media supplementation, type of culture, duration, stroma analog, and many other features. The most important differences between protocols are summarized in Table 1.

**Figure 5:**
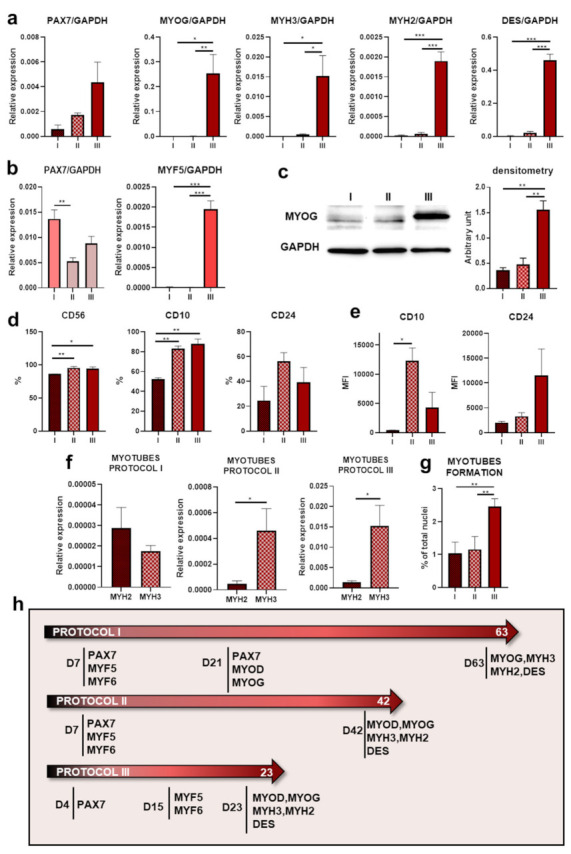
Comparison of protocols of differentiation into skeletal muscles: (**a**) evaluation of transcript levels with qPCR of *PAX7*, *MYOG*, *MYH2*, *MYH3*, and *DES* and genes at the termination of differentiation in all tested protocols, *n*: I = 2, II = 4 or 3, III = 4 or 3, * *p* < 0.05, ** *p* < 0.01, *** *p* < 0.0001; (**b**) qPCR evaluation of relative expression of *PAX7* and *MYF5* at their peaks in different protocols, *n*: I = 2, II = 4 or 3, III = 4 or 3, ** *p* < 0.01, *** *p* < 0.0001; (**c**) *MYOG* expression analysis on protein levels, representative result of Western blot at the termination of differentiation in all tested protocols with densitometry analysis, *n*: I = 2, II = 3, III = 3, ** *p* < 0.01; (**d**) differences in CD56^+^, total CD10^+^, and total CD24^+^ expression at the end of the experiment, *n*: I = 2, II = 5 or 3, III = 3, * *p* < 0.05, ** *p* < 0.01; (**e**) MFI analysis of total CD10^+^ and total CD24^+^ cells at the termination of differentiation in all tested protocols, *n*: I =2, II = 3, III = 3, * *p* < 0.05; (**f**) analysis of relative expression of *MYH2* and *MYH3* mRNA at the end day of a particular protocol, *n*: I = 2, II = 4 or 3, III = 4 or 3, * *p* < 0.05; (**g**) myotube formation represents a percent of multinucleated cells of the total nuclei; *n*: I = 2, II = 3, III = 3, ** *p* < 0.01; (**h**) schematic representation of gene expression profiles during differentiation.

Although in all of the protocols human myogenesis gene expression pattern was maintained, the levels of gene induction were different. The most important conclusion concerns the overall capacity of a particular protocol to differentiate iPS cells into skeletal muscle cells. Therefore, comparisons of the relative expression of genes in myotubes *MYOG*, *MYH3*, *MYH2,* and *DES (DESMIN)* at the end day of each protocol were performed (Figure 5a).

All four genes were significantly higher induced in cells differentiated with Protocol III. Interestingly, *PAX7* transcript was still present at the end day in all protocols, which may indicate the presence of satellite cells or the incomplete differentiation of single cells. Evaluation of *PAX7* and *MYF5* transcripts (Figure 5b) at the beginning of each protocol revealed that in all protocols, *PAX7* induction was similar, in contrast to *MYF5*. Protocol III allowed us to acquire cells with a higher level of *MYF5* expression than other protocols, which may be the reason why subsequent myotube genes also displayed higher levels in that protocol. To confirm the qPCR results, we evaluated MYOG expression on the last day of the experiment with Western blot (Figure 5c). MYOG expression was upregulated in lysate from Protocol III, which corresponds with transcript analysis. Figure 5d shows that CD56 and CD10 receptor levels were significantly lower in the cells from Protocol I, but no differences were observed between Protocols II and III. MFI analysis of total CD10^+^ population revealed that Protocol II represents the highest fluorescence signal and that the total CD24^+^ population in Protocol III had a higher fluorescence level intensity tendency (Figure 5e). Subsequently, to characterize the generated myotubes, a comparative analysis of the expression of two different myosin heavy chain types—*MYH2* and *MYH3*—was performed (Figure 5f). All protocols led to the formation of myotubes with inductions of *MYH2* and *MYH3* [2] but to various degrees. The relative levels of *MYH2* and *MYH3* expression were the highest after induction with Protocol III. Moreover, the expression of *MYH3* was higher in comparison to MYH2, confirming their fetal phenotype. Quantitative myotube formation analysis in Figure 5g shows a higher number of myotubes obtained as the result of differentiation with Protocol III. The activation of myogenic genes during protocols is summarized in Figure 5h.

The final expression pattern is similar between protocols, but as a result of differentiation with Protocol III, we obtained a population of cells with a significantly increased expression of genes that are characteristic for myotubes such as *MYOG*, *MYH2,* and *MYH3* compared to other protocols.

### 3.6. Extension of the Differentiation Protocols

**Figure 6:**
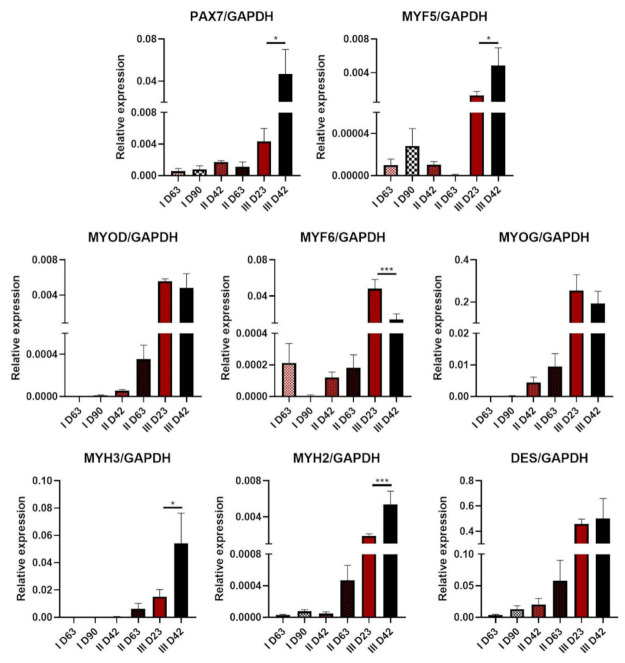
Evaluation of the effects of myogenic protocol extensions: qPCR analysis of myogenic gene expression, *n*: I = 2, II = 3, III = 3, * *p* < 0.05, *** *p* < 0.001.

To verify if the efficiency of the tested protocols can be improved by extension of the differentiation time, we cultured cells in the termination media for additional time. Protocol I was extended up to 90 days (+27 days), Protocol II to 63 days (+21 days), and Protocol III to 42 days (+19 days). Myogenic gene expression analysis was performed with qPCR (Figure 6).

Extension of differentiation time resulted in the downregulation of MYF6 expression in Protocol III. Together with the upregulation of *MYH3* and *MYH2* transcript levels, this may indicate further myotube formation and maturation. On the other hand, upregulation of *PAX7* and *MYF5* expression suggests the establishment of satellite cells or unsynchronous differentiation in the whole population. There was almost no difference in *DES* expression after the extension of Protocol III. Maintenance of cells for another 21 days in Protocol II led to subtle differences in *PAX7* and *MYF5* expression and a subtle tendency for the upregulation of *MYOD*, *MYF6*, *MYOG*, and *MYH3*, *MYH2*, *DES*. After the extension of Protocol I, *MYF5* transcript levels showed an upregulation tendency, whereas *MYF6* showed a downregulation tendency. However, the levels of relative expression were very low.

## 4. Discussion

iPS cells indisputably broaden the current knowledge of human embryogenesis regulation and allow for the development of differentiation protocols, such as differentiation into neural cells [35,36]. In the current study, we tested three different protocols of iPS cell differentiation into skeletal muscles that have been previously described in the literature to assess their efficiency. The utilization of the same cell line in different protocols allowed us to compare the effectiveness of myogenic differentiation. In all protocols, as a result of the induction of hierarchical expression of myogenic regulatory genes, we obtained elongated, multinucleated cells. Analysis of muscle-associated gene expression on the last days of differentiation suggested that Protocol III [28], based on WNT activation and TGF-β and BMP signaling inhibition, was the most efficient one in forcing myogenesis. *MYH3*, *MYH2*, *DES,* and *MYOG* genes were strongly induced. The expression of these genes indicates myotube formation and maturation [2]. Furthermore, the same tendency was observed on the MYOG protein level, consistent with the enhanced tendency of myotube formation in Protocol III. Protocol III is characterized by the application of many signaling modulators, the lack of an EB step, and the shortest duration. According to our study, restoration of accurate signaling pathways might be important in mimicking myogenesis in vitro. Apparently, when this step is recreated inefficiently, further extension of the protocol duration does not seem to compensate for proper myogenic induction. This was supported by the levels of *MYH2* and *MYH3* transcripts at the end of differentiation with Protocol I (based on EB differentiation induction, ITS addition, and selection with adhesion to collagen I type) [26]. Furthermore, in Protocol II, based on myogenic induction at the EB step with BIO, forskolin, and bFGF, levels of myogenic genes on the termination of the experiment were lower in comparison to Protocol III. This suggests that initial strong determination might not be sufficient to direct cells into muscle cells. Nevertheless, our suggestions require further verification [27].

Upregulated levels of *MYH3* in comparison to *MYH2* suggest that both Protocols II and III obtained myotubes resembling fetal skeletal muscle [2,37]. The extension of the protocols resulted in further maturation of the cells and *MYH2* transcript elevation, which characterize adult skeletal muscle [10]. The formation of EBs induces the differentiation of iPS cells, however, recent studies have suggested that this may contribute to non-specific differentiation [22]. In our study, unspecific differentiation was illustrated by flow cytometry analysis of CD10 and CD24 staining. Populations expressing exclusively the CD10 molecule are supposed to differentiate into myotubes [28]. The percentage of the CD10^+^ population in each protocol was similar and close to the half of the total population. However, total CD10 expression was significantly lower in Protocol I (56%) compared to Protocols II (75%) and III (84%). Furthermore, MFI of the total CD10^+^ population was significantly lower in Protocol I compared to Protocol II, which may suggest that sorting for CD10^+^ cells may improve Protocol II efficiency. CD24, which indicates unspecific differentiation, was present in all tested protocols. There were no differences in the percent of CD24-positive cells between tested differentiation conditions, indicating that unspecific differentiation occurs in all tested protocols. However, that observation does not reflect differences that were detected in gene expression analysis or Western blot.

Increased efficiency of Protocol III in directing iPS cells into skeletal muscles may be related to a significantly higher level of *MYF5* induction ability. Differences in *PAX7* activation between protocols suggested that all of them were able to direct iPS cells into mesenchymal progenitors. Nevertheless, further cell differentiation and myogenic induction by MYF5 activation with the inhibition of BMP and TGF-β pathways seem to be important for efficient differentiation, as has been shown before [38].

Muscle satellite cells constitute a promising element in future therapies related to the muscular system [39]. They are characterized as PAX7-positive cells [40]. At the end of differentiation in all protocols, *PAX7* expression was present. This result suggests that in this pool of cells, some of them may be muscle satellite cells. Furthermore, an additional extension of Protocol III led to the upregulation of both *PAX7* and *MYF5* while, simultaneously, *MYOD* and *MYOG* were downregulated. This may represent the establishment of satellite cells or be a result of the unsynchronous differentiation of the cells. Verification of whether these cells have other features of satellite cells requires further research in the future.

Another factor that was evaluated in our studies was the expression of vimentin. Vimentin belongs to the family of intermediate filaments and constitutes an important component of the cell cytoskeleton. Its expression is characteristic for mesenchymal cells [41]. Vimentin levels are progressively restricted during embryogenesis—it is expressed in the early stages of myogenesis, but it is downregulated at the expense of desmin, along with myogenic maturation. Furthermore, mouse myoblasts display higher levels of vimentin than myotubes [42,43]. Desmin induction was confirmed in all three protocols, and its level increases with time in Protocols I and II. Protocol III characterized a significantly higher expression of desmin, which was constant despite protocol extension in comparison to other protocols. To verify the mesenchymal phenotype of the differentiated cells and to visualize morphology, the cells were stained for vimentin on the last day of the differentiation protocols. There were no differences in the vimentin levels between protocols. There were also no differences between multinucleated and mononucleated cells in the same wells, which again confirmed the fetal character of the obtained myotubes [42]. Furthermore, vimentin staining showed that the majority of the cells obtained during differentiation had the mesodermal phenotype.

The CD56 antigen—the neural cell adhesion molecule (NCAM)—has been previously suggested as a good marker for the isolation of murine muscle progenitors [44] and described as a selective marker for human satellite cells [39]. In our study, 86% of CD56-positive cells were obtained with Protocol I, 99% with Protocol II, and 98% with Protocol III. These results question the important value of this receptor as a muscle stem cell marker. Nevertheless, CD56 may be useful for the isolation of satellite cells from adult tissue or to separate myogenic and adipogenic progenitors [45]. In our opinion, in the mixture of not-fully-committed cells, CD56 does not reflect the efficiency of skeletal muscle differentiation.

In this study, we deliberately overlooked the possibility of sorting cells in order to establish the actual differentiation potential of each tested protocol. Nevertheless, to increase the homogeny of muscle cells, the sorting of CD10^+^CD24^−^ cell populations may be applied, as proposed by Wu et al. in the original Protocol III [28].

In summary, we compared side by side three myogenic differentiation protocols. We confirmed that WNT-mediated mesodermal induction, together with the inhibition of BMP and TGF-β to induce MYF5, is the most efficient way to obtain multinucleated myotubes. We also found that CD56 is not the most specific marker to evaluate skeletal muscle differentiation efficiency. We believe that our study serves as the comprehensive comparison in the optimization of in vitro skeletal muscle cell generation for application in further studies regarding disease modeling and clinical application.

## 5. Conclusions

Our results reveal that Protocol III, based on WNT activation and TGF-β and BMP signaling inhibition in cells cultured in monolayer, is the most efficient protocol in obtaining myogenic cells. Furthermore, our results indicate that CD56 is not a specific marker for the evaluation of skeletal muscle differentiation. Our results may be significant in further studies investing the role of selected genes and pathways in both normal and pathologic myogenesis.

## Figures and Tables

**Table 1:** Comparison of myogenic protocols.

	Protocol I	Protocol II	Protocol III
**EB/monolayer**	EB and monolayer	EB and monolayer	monolayer
**WNT modulation**	-	BIO	CHIR99021
**BMP modulation**	-	-	LDN193189
**TGF-β modulation**	-	-	SB431542
**Stroma analogue**	Gelatin, Collagen I	Matrigel	Matrigel
**Passages number**	2	0	2
**Length**	63 days	42 days	23 days

## Data Availability

Data is contained within the article.
